# Effects of Oxysterols on Immune Cells and Related Diseases

**DOI:** 10.3390/cells11081251

**Published:** 2022-04-07

**Authors:** Fábio Alessandro de Freitas, Débora Levy, Cadiele Oliana Reichert, Edecio Cunha-Neto, Jorge Kalil, Sérgio Paulo Bydlowski

**Affiliations:** 1 Lipids, Oxidation and Cell Biology Team, Laboratory of Immunology (LIM19), Heart Institute (InCor), Faculdade de Medicina, Universidade de São Paulo, Sao Paulo 05403-900, SP, Brazil; fabio.alessandro@usp.br (F.A.d.F.); d.levy@hc.fm.usp.br (D.L.); kadielli@hotmail.com (C.O.R.); 2 Laboratory of Clinical Immunology and Allergy (LIM60), Heart Institute (InCor), Faculdade de Medicina, Universidade de São Paulo, Sao Paulo 05403-900, SP, Brazil; edecunha@usp.br; 3 National Institute of Science and Technology for Investigation in Immunology-III/INCT, Sao Paulo 05403-000, SP, Brazil; jkalil@usp.br; 4 Laboratory of Immunology (LIM19), Heart Institute (InCor), Faculdade de Medicina, Universidade de São Paulo, Sao Paulo 05403-900, SP, Brazil; 5 National Institute of Science and Technology in Regenerative Medicine (INCT-Regenera), CNPq, Rio de Janeiro 21941-902, RJ, Brazil

**Keywords:** oxysterols, 25-hydroxycholesterol, 7α,25-dihydroxycholesterol, immune cells, immune diseases, EBI2, LXR

## Abstract

Oxysterols are the products of cholesterol oxidation. They have a wide range of effects on several cells, organs, and systems in the body. Oxysterols also have an influence on the physiology of the immune system, from immune cell maturation and migration to innate and humoral immune responses. In this regard, oxysterols have been involved in several diseases that have an immune component, from autoimmune and neurodegenerative diseases to inflammatory diseases, atherosclerosis, and cancer. Here, we review data on the participation of oxysterols, mainly 25-hydroxycholesterol and 7α,25-dihydroxycholesterol, in the immune system and related diseases. The effects of these oxysterols and main oxysterol receptors, LXR and EBI2, in cells of the immune system (B cells, T cells, macrophages, dendritic cells, oligodendrocytes, and astrocytes), and in immune-related diseases, such as neurodegenerative diseases, intestinal diseases, cancer, respiratory diseases, and atherosclerosis, are discussed.

## 1. Introduction

Cholesterol is a vital component of cellular membranes [1,2] comprising about 20% of lipids present in plasma membrane [3,4]. Consequently, cholesterol plays a key role in maintaining the membrane integrity and fluidity, as well as having an impact on cellular physiology [3,4].

**Figure 1:**
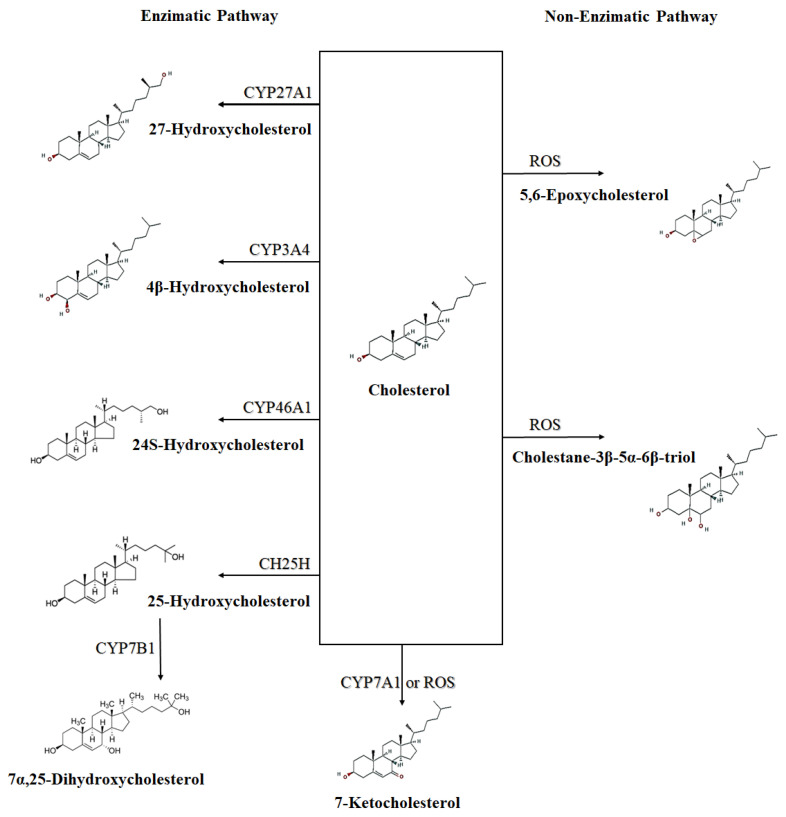
Schematic representation of enzymatic and non-enzymatic synthesis of some oxysterols. ROS—Reactive oxygen species; CYP27A1—Cytochrome P450 Family 27 Subfamily A Member 1 (sterol 27-hydroxylase); CYP3A4—Cytochrome P450 Family 3 Subfamily A Member 4; CYP7A1—Cytochrome P450 Family 7 Subfamily A Member 1 (cholesterol 7-alpha-monooxygenase); CH25H—cholesterol 25-hydroxylase; CYP46A1—Cytochrome P450 Family 46 Subfamily A Member 1 (cholesterol 24-hydroxylase); CYP7B1—Cytochrome P450 Family 7 Subfamily B Member 1 (25-hydroxycholesterol 7-alpha-hydroxylase). CYP7B1 synthetize a secondary oxysterol (7α,25-Dihydroxycholesterol) from a primary oxysterol (25-Hydroxycholesterol).

Oxysterols are oxidized derivatives of cholesterol [5,6,7,8], being intermediate compounds in the biosynthesis of bile acids, steroid hormones, and 1,25-dihydroxyvitamin D3 [5,9,10]. Oxysterols can be formed either enzymatically, by the action of some members of the CYP (cytochrome P450) family, or non-enzymatically, by the action of ROS (Figure 1) [3,9,10]. Oxysterols present in the diet may contribute to the total pool of oxysterols in the body [9,10].

Oxysterols can also be classified into those synthesized directly from cholesterol, the primary oxysterols, which are formed by side-chain changes (such as 24S-, 25-, (25R)-26 and 27-hydroxycholesterols), and by ring changes (which includes 7α-hydroxycholesterol and 7β-hydroxycholesterol); and those derived from primary oxysterols, the secondary oxysterols, such as 7α,25-dihydroxycholesterol (Figure 1) and 7α,(25R)-26-dihydroxycholesterol, which are generated from 25-hydroxycholesterol and (25R)-26-hydroxycholesterol, respectively [11].

Although oxysterols are metabolic intermediates, several of them are bioactive, and their absence or excess can significantly affect the pathophysiology of some diseases [5]. Indeed, different oxysterols can have different actions, depending on their concentration and the type of cell or tissue, from changes in gene expression and lipid metabolism [12] to promotion of cell death, proliferation or differentiation [13,14,15,16,17,18,19]. In this way, some oxysterols have effects on several cells of the immune system, contributing to the development of several diseases. In the following sections the effects of some oxysterols on immune cells and related diseases will be discussed.

Oxysterols such as 7-ketocholesterol, 7β-hydroxycholesterol, 24-hydroxycholesterol, cholestane-3β,5α, 6β-triol, and mainly 25-hydroxycholesterol, have proinflammatory properties, stimulating THP-1 (human monocyte-derived macrophages) cells, and porcine retinal pigment epithelial cells, to produce IL-8 [20], involving the MEK/ERK1/2 cell signaling pathway [21]. In addition, when U937 (human promonocytic leukemia cells) and THP-1 cells are in the presence of oxysterols, mainly 7β-hydroxycholesterol and 25-hydroxycholesterol, these cells secrete several chemokines involved in the recruitment of immunocompetent cells at the subendothelial level such as MCP-1, MIP-1β, TNF-α, IL-1β and IL-8 [21]. 25-Hydroxycholesterol can potentiate LPS-induced IL-1β secretion in human mononuclear cells, principally under hypoxic conditions [22].

The ageing process promotes increasing vulnerability to major pathologies such as atherosclerosis, Alzheimer’s disease, age-related macular degeneration, cataracts, and osteoporosis [23]. The inflammation process increases during ageing, and it is linked to mitochondrial dysfunction and an increase in ROS production [24]. Oxysterols can induce severe dysfunctions in cells organelles, especially mitochondria, and can play a key role in the control of cell death induction, inflammatory status, redox homeostasis, and lipid metabolism, contributing to explain the participation of these molecules in ageing processes and in age related diseases [23]. 7-Ketocholesterol and 7β-hydroxycholesterol exert a strong effect on mitochondrial activity contributing to cell degeneration [25]. The induced mitochondrial depolarization caused by 7-ketocholesterol and 7β-hydroxycholesterol involve PDK1/PKB (Akt)/GSK3 metabolic pathways [26,27]. These alterations caused on mitochondria by oxysterols on mitochondria can be the core of several age-related diseases [23].

## 2. Oxysterols and the Immune System

The plasticity of immune cells has many implications in the pathogenesis and resolution of several diseases, such as chronic inflammatory disorders, metabolic disorders, cancers, and autoimmune diseases [28]. It is known that nutritional status and metabolic disorders such as obesity can influence the immune response, as immune cells can interact with different lipids, which can affect the plasticity of cells such as macrophages and T lymphocytes [28]. Pathways that promote lipid synthesis and accumulation tend to drive a proinflammatory phenotype, while pathways that enhance β-oxidation and lipid efflux tend to drive immune cells toward an antiinflammatory phenotype [28]. As examples: (a) in lean adipose tissue, CD4+ T cells and M2 macrophages express antiinflammatory phenotypes; (b) in overnutrition or obesity, there is an elevated level of saturated fatty acids, which drives the regulation of the influx and activation of inflammatory macrophages (M1) and lymphocytes (Th1, CTL and Th17) in adipose tissue; (c) in cardiovascular disease, there is the presence of both M1 and M2 macrophages; macrophages can infiltrate the arteries and engulf oxysterols, giving rise to foam cells; (d) the liver has antiinflammatory immune cells such as M2-associated Kupffer cells and CD4+ T cells (Th2 and Treg); in fatty liver disease, there is an elevate level of lipids, which drives the recruitment of inflammatory monocytes (Ly6C^hi^) and its differentiation into M1 macrophages [28].

There is a connection between immune system signaling and oxysterols [3]. Oxysterols acts in the regulation of the adaptive immune system [3,29] and have important roles in the innate immune system, such as the direct regulation of the inflammatory programming and participation in the development of immune response [30,31] and signaling [3,29]. Oxysterols and their receptors can regulate and modulate the function and phenotype of immune cell subsets such as macrophage, B and T cells, neutrophils, and dendritic cells [28,32].

In fact, according to some authors, some oxysterols could also be called “immunosterols”, due to their role in the immune system [3].

## 3. Oxysterol Receptors: LXR and EBI2

Oxysterols are signaling mediators that act in several membrane and nuclear receptors, including estrogen receptor α, and retinoic acid receptor-related orphan receptors. The most important, in terms of the immune system, are the liver X receptors (LXRs) pathway [33] and the G-protein-coupled receptors (GPCRs) EBI2 [32].

### 3.1. LXR

**Figure 2:**
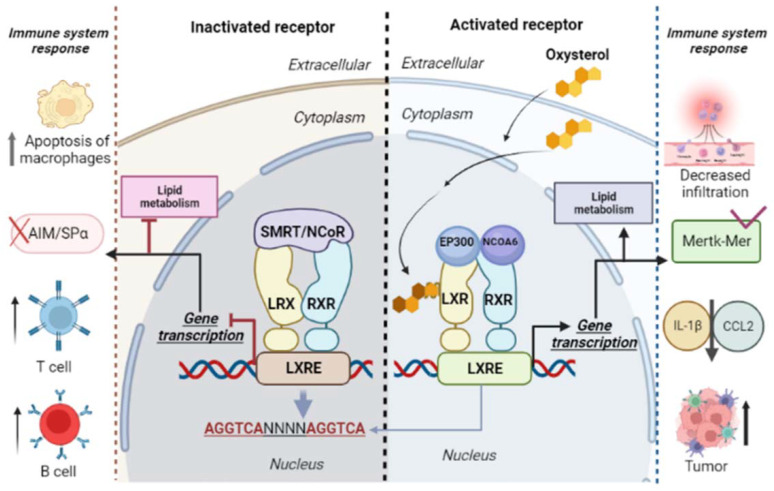
Liver X receptors (LXRs) and retinoid X receptors (RXRs) form heterodimers and bind to LXR response elements (LXREs). The LXR/RXR binding regions are composed of repeated sequences of AGGTCA and four nucleotides (NNNN). This region is responsible for the regulation of activated target genes via LXR/RXR. Inactivation of the LXR/RXR complex occurs by binding with corepressors (nuclear receptor corepressor (NcoR), retinoic acid silencing mediator, and thyroid hormone receptor (SMRT)). In the presence of oxysterols, coactivators (nuclear receptor coactivator 6 (NCOA6) and histone acetyltransferase p300 (EP300)) bind to the LXR/RXR complex, activating the expression of genes involved in lipid metabolism and in innate and cellular immune response. Both the inactivation and the activation of LXR receptors are associated with immune responses.

Oxysterol LXR-dependent activity has a biological influence on immune cells in different pathological contexts, such as infectious diseases, autoimmune diseases, and cancer [34,35]. Oxysterols can bind to LXR receptors from immune cells, modulating their actions [12,32] (Figure 2). Liver X receptors (LXRs) are ligand-activated transcription factors that belong to a superfamily of 48 ligand-dependent transcription factors. There are two LXR isoforms: (1) LXRα—nuclear receptor subfamily 1 group H member 3 (NR1H3); and (2) LXRβ—nuclear receptor subfamily 1 group H member 2 (NR1H2) [34,36,37,38]. Both, LXRα and β, as heterodimers, complex with the retinoid X receptor (RXR), a common partner for several nuclear receptors such as the peroxisome proliferator-activated receptors (PPARs). The LXR/RXR complex links to a LXR response element (LXRE) in the promoter region of target genes, promoting the regulation of gene expression through mechanisms that include direct activation, ligand-independent or ligand-dependent repression, and trans-repression [39].

LXR signaling, mainly LXRβ, inhibits the proliferation of T and B cells. The activation of T and B cells triggers mechanism for cell proliferation: the induction of the sulfotransferase family 2B member enzyme (1SULT2B1), which inactivates oxysterols as LXR ligands by a sulfation process; and the promotion of sterol regulatory element-binding protein (2SREBP-2) pathway for cholesterol synthesis [35].

Oxysterols, in an LXR-independent manner, can recruit protumor immune cells within the tumor microenvironment [32]. However, these chemoattractant tumor-derived oxysterols (e.g., 22R-hydroxycholesterol and 25-hydroxycholesterol) also activate LXRs, inhibiting the CC chemokine receptor-7 (CCR7) expression in maturing dendritic cells, and impairing their migration to draining lymph nodes [40].

In addition, oxysterols such as 24S-hydroxycholesterol can induce apoE-mediated cholesterol efflux in astrocytes via an LXR-controlled pathway, a relevant process in chronic and acute neurological diseases [41]. Interestingly, 24S-hydroxycholesterol is also involved in the suppression of synaptic vesicle exocytosis during 20 Hz activity at the neuromuscular junctions, this action being dependent on both LXR activation and upregulation of NO-signaling [42]. These findings show how LXR/oxysterol studies are important for clarifying the development of some diseases.

In contrast with the traditional knowledge of LXRs’ action on cell nucleus, these receptors can also have non-nuclear functions such as: LXRβ action on endothelial cells caveolae/lipid rafts that entails crosstalk with ERα, which promotes NO production and maintains endothelial monolayer integrity in vivo [43]; LXRβ expression in platelets (anucleate), being responsible for the inhibition of platelet function and thrombosis [44]; 25-hydroxycholesterol oxidant capacity and its regulatory action on synaptic vesicle mobilization via the activation of lipid raft-associated LXRs could trigger signaling via estrogen receptor α-Gi-protein-Gβγ-phospholipase C-Ca^2+^-protein kinase C pathway [45].

### 3.2. EBI2 (GPR183)

The orphan seven-transmembrane G-protein-coupled receptor 183 (GPR183), also called Epstein–Barr-virus-induced molecule-2 (EBI2) [46,47,48,49], was identified in 1993 by Birkenbach et al. as one of the principal genes induced by Burkitt’s lymphoma cell line BL41 when these cells are infected by the Epstein–Barr virus (EBV) [47,50]. In 2011, Hannedouche et al., and Liu et al. reported that the oxysterol 7α,25-dihydroxycholesterol is a potent and selective EBI2 agonist and its most likely endogenous ligand [51,52]. EBI2 is coupled with Gαi, and ligand engagement leads to the activation of RHO family GTPases and mitogen-activated protein kinases such as the extracellular signal-regulated kinase (ERK) and P38 to intracellular calcium flux [30].

**Figure 3:**
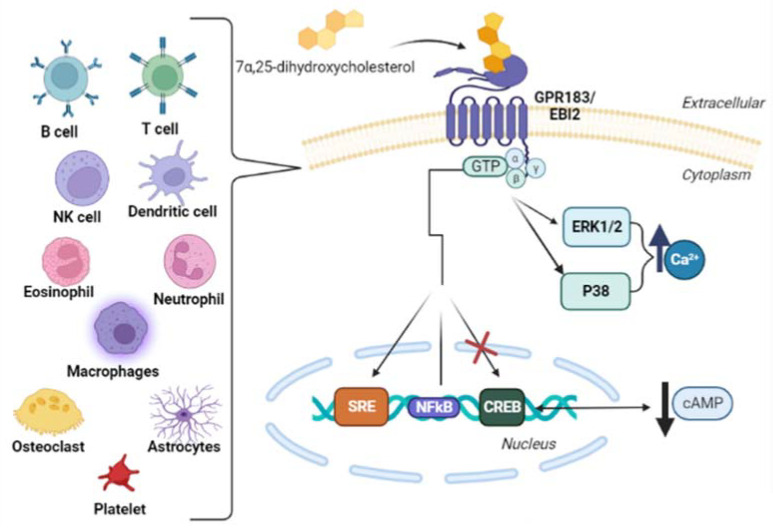
Schematic pathway of EBI2 receptor activation in cells that express this receptor. 7α,25-dihydroxycholesterol, a ligand of EBI2 (Epstein–Barr-virus-induced molecule-2), activates the GTPases family and mitogen-activated protein kinases/extracellular signal-regulated kinase (ERK) and P38 to intracellular calcium flux. In the nucleus, GTPases stimulate the expression of SRE (Serum Response Element) and the expression of NFκB (nuclear factor kappa-light-chain-enhancer of activated B cells) (weak stimulation), and inhibit the expression of CREB (cAMP Response Element-Binding Protein), downregulating the production of cAMP (cyclic Adenosine Monophosphate) [53,61].

Although the EBI2 expression was firstly identified in B cells, it is now known that EBI2 is expressed in several cells such as natural killer cells [52,53], B cells [51,52,53,54], T cells [52,53], monocytes/macrophages [52,53,54,55], dendritic cells [51,52], eosinophils [53,56], platelets [57], osteoclasts [55], and neutrophils [52]. In addition, EBI2 expression has also been characterized in astrocytes [58] and in the early developmental stages of immune cells, including hematopoietic stem and progenitor cells and thymocytes [59,60]. Figure 3 shows the EBI2 receptor and its activation pathway.

EBI2 is responsible for regulating the positioning of immune cells in secondary lymphoid organs. Polymorphisms in this receptor have been associated with some inflammatory autoimmune diseases [51], such as type 1 diabetes [54]. Furthermore, EBI2 participates in: the stimulation of migration of B, T and dendritic cells, monocytes [62], and astrocytes; the increase of B cell proliferation; and in the negative regulation of type I interferon in dendritic cells and monocytes [62,63]. EBI2 deficiency can result in reduced early immunoglobulin-M (IgM) and IgG antibody response to a T cell dependent antigen, in addition to interfering with the B cell migration to the outer follicular niche in the spleen and lymph nodes [46]. In fact, EBI2 also represents another key chemotactic receptor, together with CXCR5, CXCR4, and CCR7, directing B cells migration within secondary lymphoid tissues [62].Chemoattractant receptors of the GPR family have essential roles in coordinating the migration of lymphocytes to produce an efficient response against microorganisms [54], such as in the spleen, where EBI2 plays an important role controlling the immune cell migration during the course of a T-dependent antibody response [47]. However, when these receptors are dysregulated, an initiation or progression of inflammatory and autoimmune disorders can occur [54]. Polymorphisms in the gene encoding EBI2 have been associated with inflammatory processes. In addition, EBI2 was shown to be involved in the regulation of the inflammatory response of macrophages in rats [54].

The dysregulation of EBI2 expression has also been related to human neoplastic diseases, such as acute myeloid leukemia, chronic lymphocytic leukemia, diffuse large B cell lymphoma, and follicular and germinal center B-like diffuse large B cell lymphoma [64].

## 4. Main Oxysterols That Are Importance to the Immune System

### 4.1. 25-Hydroxycholesterol

25-Hydroxycholesterol has been described as promoting inflammatory and anti-inflammatory effects [3]. Cholesterol 25-hydroxylase (CH25H) is the enzyme responsible for oxidizing cholesterol to form 25-hydroxycholesterol [65]. CH25H does not belong to the cytochrome P450 (CYP) family; it is a member of a family of enzymes that uses diiron cofactors to catalyze hydroxylation [65].

25-Hydroxycholesterol plays a key role in the regulation of B cells. 25-Hydroxycholesterol enhances the expression of IL-8, IL-6 and macrophage colony-stimulating factor (MCSF) and inhibit the production of IL1-β by inhibiting the Sterol Regulatory Enhancer Binding Protein (SREBP) [66]. The inhibition of SREBP also modulates sterol pathway flux resulting in the formation of STING/cGAMP complex that phosphorylates TBK, which further phosphorylates IFR3 to activate the expression of IL1-β [3]. 25-Hydroxycholesterol also induces the production and release of cytokine C-C Motif Chemokine Ligand 5 (CCL5) [66]. CCL5 is a pro-inflammatory cytokine involved in the recruitment of cells to the site of infection, thus amplifying the immune response and inflammation [3].

The *CH25H* gene belongs to the family of interferon-stimulating genes (ISGs), which play key roles in inflammation, innate immunity, and subsequent adaptive immune responses through interferon signaling [67]. Inflammatory mediators promote the upregulation of *CH25H* in dendritic cells and macrophages; this indicates that 25-hydroxycholesterol has a potential function in innate immune regulation [68]. In addition, 25-hydroxycholesterol and 7α,25-dihydroxycholesterol are synthesized and secreted by macrophages [69,70]

An infection promoted by virus or bacteria leads to the production of type I interferon, rapidly inducing *CH25H* to generate 25-hydroxycholesterol [71]. The increase in 25-hydroxycholesterol production has also been observed in the lung during *M. tuberculosis* infection [70] and in acute lung injury models [72]. In addition, in its role as an anti-inflammatory molecule, 25-hydroxycholesterol blocks the activation of SREBP, which regulates cholesterol biosynthesis and inflammasome activity, as stated above [71]. Specifically, 25-hydroxycholesterol has been shown to decrease inflammasome activity via NLRP3, and subsequent IL-1β production [71].

### 4.2. 7α,25-Dihydroxycholesterol

CH25H and CYP7B1 (25-hydroxycholesterol 7-alpha-hydroxylase) are the 2 enzymes involved sequentially in the main route of 7α,25-dihydroxycholesterol synthesis from cholesterol [30,73]: 25-hydroxylation of cholesterol by CH25H, followed by 7α-hydroxylation by CYP7B1 [65,74] (Figure 1). In addition, CYP3A, CYP27A1, and CYP46A1 enzymes also have sterol 25-hydroxylation activity [65]. An alternative synthesis route for 7α,25-dihydroxycholesterol is via 7α-hydroxylation of cholesterol by CYP7A1 followed by 25-hydroxylation of 7α-hydroxycholesterol (7α-HC) [51]. Another enzyme, HSD3B7, is responsible for 7α,25-dihydroxycholesterol metabolization to a 3-oxo derivative [30,73]. CH25H, CYP7B1, and HSD3B7 are also involved in the control of EBI2-ligand concentration in lymphoid tissues and are an extrahepatic pathway that regulates oxysterol production in these tissues [73].

Oxysterols are ligands (orthosteric or allosteric) for G-protein-coupled receptors (GPCRs): EBI2, C-X-C Motif Chemokine Receptor 2 (CXCR2), G-protein-coupled receptor 17 (GPR17), and the Smoothened receptor [75,76,77,78].

EBI2 and key enzymes involved in 7α,25-dihydroxycholesterol synthesis are highly regulated during inflammation and could be involved in autoimmune diseases, cardiovascular diseases, neurodegenerative diseases, some metabolic diseases (dyslipidemia, obesity, and diabetes), in addition to some types of cancer and inflammation [62].

## 5. Oxysterols and Immune System Cells

Oxysterols can influence several immune system cells, changing their functions, such as immunoglobulin production, formation of neutrophil extracellular traps, and differentiation and migration of lymphocytes [79].

### 5.1. B Cells

B cells undergo a series of migratory events, which guide them to the appropriate microenvironment for the adequate activation and differentiation [64].

7α,25-dihydroxycholeserol is a EBI2 ligand, a cellular receptor expressed by B cells. The expression of EBI2 is increased when B cells are activated and downregulated in the germinal center, the specialized microstructure formed in secondary lymphoid tissues, responsible for producing long-lived antibody-secreting plasma cells and memory B cells [46]. In addition, EBI2 is responsible for mediating the correct location of B cells in humoral immune responses [48]. The overexpression of EBI2 promotes the localization of B cells in the outer follicle which are highly specialized histological structures [46].

EBI2 plays a key role in the regulation of B cell migration during immune activation and response [46,62,80]. Just a few hours after encountering the antigen, EBI2 is responsible for the migration of B cells to the external follicles of lymphoid tissue in the region where the antigen enters the structure [30]. Th cells send CD40 signals, which sustain EBI2 expression on activated B cells while promoting CCR7 downregulation, which leads to EBI2-dependent B cell migration to the outer follicle, but now with a preference for interfollicular regions [30]. During differentiation into germinal center B cells, downregulation of EBI2 has an important role for precursor cell migration to the follicle center. Downregulation of EBI2 may occur due to B cell lymphoma 6 (BCL6)-mediated repression, and may involve signaling through signal transducer and activator of transcription 6 (STAT6)-dependent cytokine receptors. However, the expression of low levels of EBI2 can be maintained in some germinal center B cells, such that the receptor can potentially influence cells even in this compartment [30].

The HSD3B7 enzyme, produced by stromal cells, are responsible for inactivating 7α,25-dihydroxycholesterol. However, the absence of this enzyme causes an increase in the availability of EBI2-ligand, resulting in the failure of B cell positioning. The same mechanism that controls the EBI2-ligand production by the HSD3B7 enzyme is observed in dendritic cells. Therefore, considering the crucial role that CYP7B1, CH25H and HSD3B7 enzymes have in the maintenance of an adequate EBI2-ligand gradient, decreased production of these enzymes could be associated with impaired humoral immune responses [73].

### 5.2. T Cells

The T cell-dependent humoral immune response is highly linked to EBI2. Dysregulation of this receptor contributes to B cell diseases such as diffuse large B cell lymphomas and chronic lymphocytic leukemia [61]. In these cases, EBI2 expression is downregulated while it is upregulated in post-transplantation lymphoproliferative disorders [61].

A subset of T CD8+ cells and T CD4+ cells also express EBI2 and are responsive to 7α,25-dihydroxycholesterol, which promotes migratory process of these cells [52]. For example, 7α,25-dihydroxycholesterol promotes the migration of activated CD4+ T cells to tissues with inflammation in experimental autoimmune encephalomyelitis through its interaction with EBI2 [81].

7β,27-dihydroxycholesterol is a selective activator of nuclear receptor RAR-related orphan receptor γ (RORγ). In addition, RORγ is the master nuclear transcription factor needed for Th17 differentiation, which is affected by changes in the enzymes related to 7β,27-dihydroxycholesterol [82].

The nuclear receptor RAR-related orphan receptor γ t (RORγt) is required for the generation of IL-17-producing CD4+ Th17 cells, which are essential for hosting defense, as they are also involved in the development of autoimmune diseases [82]. Soroosh et al., (2014) identified the 7β,26-dihydroxycholesterol as the most potent and selective activator for RORγt. Both 7β,26-dihydroxycholesterol and its isomer 7α,26-dihydroxycholesterol can promote the differentiation of murine and human IL-17 producing Th17 cells in a RORγt-dependent manner [82]. These findings could contribute to a new design for RORγt modulators, and also provide new targets for inhibiting IL-17 production [82]. In addition, RORα (NR1F1), RORγ (NR1F3), NR2F6, and a ligand-regulated PAS superfamily member, Ahr, play important roles during the differentiation of Th17 inflammatory T cells [83].

### 5.3. Macrophages

Macrophages are functional cells that play central roles in both innate and adaptive immunity and homeostasis [84,85]. There are differences in mature macrophages from each tissue, due to their high sensitivity to microenvironments, therefore differing in their morphology, expression of cell surface receptors, secretome, and functional capabilities [86].

Three important aspects of macrophage biology are regulated by oxysterols: (1) regulation of lipid transport and metabolism; (2) inflammatory responses; (3) cytotoxicity [85]. In addition, LXRs, insulin-induced genes (Insigs), and oxysterol-binding protein (OSBP)/OSBP-related protein (ORP) family members have been identified as key acceptors for these functions of oxysterols [85]. In addition, activated macrophages are classified into: M1 (pro-inflammatory), which are polarized in infections by pathogenic microorganisms, IFN-γ, and/or TLR ligands; and M2 (anti-inflammatory), which are activated by IL-4 or IL-13 [87]. In this way, Krüppel-like factor 4 (KLF4) in the macrophage favors the transition from the pro-inflammatory M1 to anti-inflammatory M2 macrophage phenotype [88]. Interestingly, Lamtor1, v-ATPase and mTORC1 integrate the intracellular amino-acid sufficiency signal and the extrinsic IL-4 signal, leading to the production of 25-hydroxycholesterol and subsequent activation of LXR, ultimately resulting in the polarization of M2 macrophages [87]. In addition, 27-hydroxycholesterol can also stimulate M2 macrophage polarization toward the immunomodulatory functional phenotype [89]

Macrophages can produce and secrete high amounts of 25-hydroxycholesterol in response to the activation of Toll-like receptors [68,90]. However, its expression is notably silent in quiescent immune cells [68].

Interferon (IFN) regulates the gene *CH25H*, at least in rats. This is responsible for synthesizing 25-hydroxycholesterol in macrophages, after IFN-stimulation or viral infection, in this way acting as a potent paracrine inhibitor of viral infection for different viruses. This response to IFN is via the direct recruitment of signal transducer and activator of transcription 1 (Stat1) to the promoter proximal region of the *CH25H* gene. This mechanism shows the importance of Ch25h in the innate immune pathway [69]. In addition, the Stat1 binding to the *CH25H* promoter provides a critical molecular link between innate immune stimulation, infection, and the secretion of 25-HC by macrophages [69]. By using transcriptional regulatory-network analyses, genetic interventions and chromatin immunoprecipitation experiments, Blanc et al., (2013) have shown that Stat1 is strongly linked to *CH25H* regulation to IFN in macrophages [69].

24,25-Epoxycholesterol and 25-hydroxycholesterol are ligands for the LXR [91]. Activation of LXR by 24,25-epoxycholesterol enhances the expression of genes encoding Abca1 and Abcg1 transporters that mediate cholesterol efflux, and inhibit the expression of inflammatory response genes [92].

Diczfalusy et al., (2009) [66] have shown that CH25H is strongly upregulated by lipopolysaccharide (LPS). The injection of LPS into healthy volunteers increased 25-hydroxycholesterol concentration in their plasma [66]. In addition, Kdo2-lipid A, the active component of an inflammatory LPS, acts as a Toll-like receptor 4 (TLR4) agonist, and promotes a 4-fold increase of *CH25H* mRNA expression on RAW264.7 cell line (mouse macrophage) [90], while the increase of *CH25H* mRNA in bone marrow-derived macrophages has been reported to be 15-fold [68].

In addition, Ngo at al., (2022) [70] have described an upregulation of the CH25H enzyme and CYP 7B1 in alveolar and infiltrating macrophages of dysglycemic mice lung infected by *M. tuberculosis* even with elevated in 25-hydroxycholesterol levels. This finding was linked with the increased EBI2 expression, responsible for the oxysterol-mediated recruiting of immune cells to the lung [70]. Barlett et al., (2020) [63] have shown that EBI2 activation by 7α,25-dihydroxycholesterol reduces both *M. tuberculosis* and *M. bovis* growth in primary human monocytes, via the reduction of IFN-β and IL-10 expression and enhanced autophagy [63].

In a lipidomic analysis using mouse macrophage RAW264.7 (RAW) activated by Kdo2-lipid A (KDO), the active component of LPS, a 3-fold increase in intracellular 25-hydroxycholesterol concentration was observed, with a 4-fold increase in *CH25H* mRNA expression [90,93]. A total of 24 h after stimulation, both cellular cholesterol and 24S,25-epoxycholesterol levels had doubled [90]. The increase in both cellular cholesterol and 24S,25-epoxycholesterol levels involves LPS stimulation of the complex 1 of the mammalian targets of rapamycin (mTORC1), leading to the activation of the SREBP-2 pathway [94,95].

The stimulation of macrophage Toll-like receptor 4 (TLR4) increases *CH25H* expression and 25-hydroxycholesterol synthesis. This leads to the suppression of interleukin-2 (IL-2), which mediates the stimulation of B cell proliferation, inhibits the activation of cytidine deaminase (AID) expression, leading to a decreased IgA production [68]. The IgA suppression by B cells in response to TLR activation is responsible for a mechanism that negatively regulates the local and systemic adaptive immune response by the innate immune system [68].

In macrophages, the connection between LXR and TLR signaling is TLR3- and TLR4-mediated, and the IRF3-dependent activation of macrophages blocks the induction of LXR target genes and inhibits cholesterol efflux [96]. In contrast, TLR3 and TLR4 agonists induce CH25H and, consequently, oxysterol synthesis. Therefore, it is possible that immune modulation can be accompanied by LXR target gene induction and enhanced cholesterol efflux [12].

LXR activation in macrophages, mediated by oxysterols, promotes macrophage survival and suppresses TLR-mediated innate immune responses, characterized by the reduced production of IL-6, IL-1β, MCP-1, MCP-3, iNOS, COX-2, and MMP-9 [97].

Activated macrophages in mice, in the absence of IFN-stimulation of the *CH25H* gene, are unable to produce 25-hydroxycholesterol, and overproduce inflammatory interleukin-1 (IL-1)-family cytokines. 25-Hydroxycholesterol is an antagonist of SREBP, reducing Il1β transcription and repressing IL-1–activating inflammasomes. Therefore, 25-hydroxycholesterol is a critical mediator in the negative-feedback pathway of IFN signaling on IL-1-family cytokine production and inflammasome activity [71].

Type 1 IFN restrains IL-1β driven inflammation in macrophages by upregulating *CH25H* and 25-hydroxycholesterol and repressing the sterol-sensing transcription factor SREBP2-driven cholesterol synthesis. In the absence of CH25H, cholesterol overload triggers mitochondrial DNA release and activation of AIM2 inflammasomes in activated macrophages [98]. Therefore, the anti-inflammatory action of 25-hydroxycholesterol in activated macrophages maintains mitochondrial integrity and prevents the AIM2 inflammasome activation [98].

### 5.4. Dendritic Cells

Similar to macrophages, *CH25H* is upregulated in dendritic cells in response to cell surface TLR4 activation by LPS and intracellular TLR3 ligands [12]. In addition, TLR-mediated expression of *CH25H* is dependent on TIR-domain-containing adapter-inducing interferon-β (TRIF), production of type I interferon (IFN-1), and signaling through the IFN α/β receptor/Janus kinase/Signal transducer and activator of transcription 1 (IFNR/JAK/STAT1) pathway. In addition, *CH25H* is an IFN-responsive gene in dendritic cells, as in macrophages, during innate immune responses, and the early expression of *CH25H* implies a role for oxysterols in the regulation of innate immunity [12].

Tumor-derived oxysterols such as 22(R)-hydroxycholesterol and 27-hydroxycholesterol can exert opposite effects on the expression of CCR7 in dendritic cells. The different action of oxysterols on dendritic cells is related to the differentiation stage of these cells (immature versus mature), possibly through the differential activation of LXRα and/or LXRβ isoforms [34]. In addition, the stimulation of LXR in dendritic cells during innate response activation decreases the production of CD86 and IL-12, enhances the secretion of IL-10, and blocks the activation of T cells [99].

### 5.5. Oligodendrocytes

Oxysterols have been shown to promote adverse effects on oligodendrocyte viability in vitro. The treatment of 158N (oligodendrocyte) cell line with 25-hydroxycholesterol or 22(S)-hydroxycholesterol induce cell death and morphological changes independently of LXR signaling [100]. In addition, the presence of oxysterol biosynthetic enzymes and oxysterols has been shown in oligodendrocytes, indicating that oxysterols may signal in an autocrine/paracrine manner in these cells [100].

### 5.6. Astrocytes

Oxysterols play important roles in astrocyte biology. For instance, they inhibit astrogliosis and there is a correlation between cholesterol synthesis in the central nervous system and reactive astrocyte proliferation. Human and mouse astrocytes express EBI2 as well as the enzymes necessary for the synthesis and degradation of 7α,25-dihidroxycholesterol, CH25H, CYP7B1 and HSD3B7. The receptor expressed in astrocytes is functional and signals via ERK1/2 phosphorylation and Ca^2+^ influx. In this way, EBI2/oxysterol signaling is involved in normal myelin development as well as in the release of proinflammatory cytokines and communication with macrophages [101].

## 6. Oxysterols and Immune System-Related Diseases

Oxysterols are involved in the control of several physiological and pathologic processes [13,14,15,16,17,18,19,32] including, as we have described, the modulation of immune responses. Therefore, they are also involved in several diseases in which the immune system plays an important role [11,79].

### 6.1. Neurodegenerative Diseases

24-Hydroxycholesterol is the predominant metabolite of brain cholesterol, and this oxysterol is involved in neurodegenerative diseases such as multiple sclerosis [102]. In addition, 24-hydroxycholesterol can also modulate the atrial β-adrenoceptor signaling, contributing to the regulation of contractility [103]; moreover, 24-hydroxycholesterol can potentiate and selectively enhance N-methyl-D-aspartate receptors’ (NMDA) function at a site distinct from other modulators [104].

Alterations in cholesterol homeostasis are being implicated in the pathogenesis of amyotrophic lateral sclerosis (ALS), followed by several oxysterols, bile acids and auto-oxidized sterols, that are increased in the lumbar SC, plasma, and feces during disease development [105]. Lipid raft disruption, likely due to ceramide accumulation, could be one of the earlier events of ALS, which may trigger neuromuscular abnormalities. 25-Hydroxycholesterol can restore the membrane and functional properties of neuromuscular junctions at the early stage of disease [106] or, contradictorily, 25-hydroxycholesterol could be actively involved in the ALS pathogenesis, with the involvement of the GSK3-ß activation and neuronal apoptosis [107]. In addition, in ALS it is hypothesized that oxysterols can be used as biomarkers, particularly 25-hydroxycholesterol and 24S-hydroxycholesterol, as well as phytosterols such as β-sitosterol and its glucoside derivatives [108]. In ALS, a impaired activity of the CYP27A1 enzyme is observed, leading to a failure of the central nervous system to remove the cholesterol excess that may be toxic to neuronal cells, compounded by a reduction in neuroprotective 3β,7α-diHCA and other LXR ligands [109]. 24S-hydroxycholesterol can suppress the exocytotic release of neurotransmitters in response to intense activity via NO/lipid raft-dependent pathway in the neuromuscular junctions of SODG^93A^ mice [110]. Considering the relation between ALS and LXRs, an association was observed between SNP genotypes of the LXRα gene and late age of onset in ALS patients. In addition, the C/C genotype of SNP rs2695121 from the LXRβ gene is associated with a 30% increase in the rate of survival for ALS [111].

The major mechanism of cholesterol removal from the brain is its hydroxylation in C24 by the CYP46A1. In this way, CYP46A1 can be associated with different neurologic diseases, such as Alzheimer’s disease and Dravet and Lennox–Gastaut syndromes [112]. Changes in CYP46A1 activity in the brain can lead to altered levels of 24-hydroxycholesterol, changing the modulation of LXRs and N-methyl-D-aspartate receptors (NMDARs) [112]. The cholesterol turnover of the brain is dependent on CYP46A1 activity and 24-hydroxycholesterol production [113]. In addition, 24S-hydroxycholesterol could be involved in several neurodegenerative diseases such as Alzheimer’s disease, Huntington’s disease, Parkinson’s disease, multiple sclerosis and amyotrophic lateral sclerosis [114].

#### 6.1.1. Multiple Sclerosis

Multiple sclerosis is a common autoimmune disease involving the nervous system [115]. Multiple sclerosis patients have altered oxysterol levels both in blood and in cerebrospinal fluid, compared to healthy controls, including 24(S)-hydroxycholesterol, the predominant metabolite of brain cholesterol [11].

24-Hydroxycholesterol, (25R)-26-hydroxycholesterol, and 7α-hydroxycholesterol levels are lower than in healthy individuals. In addition, 7-Ketocholesterol levels are higher in progressive multiple sclerosis patients compared with multiple sclerosis relapsing–remitting [116,117].

The concentrations of 7-ketocholesterol detected in the cerebrospinal fluid of multiple sclerosis patients are capable of inducing neuronal damage by the activation and migration of microglial cells [118]. This pathway involves the 7-ketocholesterol entering in the microglia nucleus, resulting in the translocation of the nuclear factor kappa B (NF-κB) and the activation of poly(ADP-ribose)-polymerase 1(PARP-1), followed by the expression of inducible nitric oxide synthase (iNOS), and migration-regulating integrins, such as CD11a and intercellular adhesion molecule 1 (ICAM-1). This mechanism links the demyelination process with the progressive neuronal damage [118].

In the spinal cord of mice with experimental autoimmune encephalomyelitis, a model for multiple sclerosis studies, there is an enhanced concentration of 25-hydroxycholesterol, 7α,25-dihydroxycholesterol, 7α,26-dihydroxycholesterol, and 7α,24-dihydroxycholesterol. However, decreased concentrations of 24-hydroxycholesterol and 26-hydroxycholesterol were observed [74]. In addition, there are reports that multiple sclerosis patients had lower plasma levels of cholesterol and 25-hydroxycholesterol than healthy people [83,119]. It has been suggested that a lower cholesterol level favors Th17 polarization and hence the subsequent development of Th17 inflammatory cell-dominant autoimmune diseases such as multiple sclerosis [83,119].

Genetic analysis of multiple sclerosis patients revealed a potential association between variants of the cholesterol 25-hydroxylase (*CH25H*) gene and primary progressive multiple sclerosis [120]. Moreover, genetic variants in NR1H3 (LXRα) were also found to be associated with an increased risk of developing progressive multiple sclerosis [121].

In the evolution of experimental autoimmune encephalomyelitis in mice, there is an increase in the expression of *CH25H* by microglia and CYP7B1 by central-nervous-system-infiltrating immune cells. These events in the central nervous system elevate the concentration of 7α,25-dihydroxycholesterol, interleukin-23 (IL-23), interleukin-1 beta (IL-1β) (critical pro-inflammatory cytokines), and interleukin-1β (IL-1β), maintaining the expression of EBI2 in differentiating T helper 17 (Th17) cells [74]. 

EBI2 enhances the early migration of encephalitogenic T cells into the central nervous system in a transfer experimental autoimmune encephalomyelitis model [74]. In addition, there are abundant EBI2-expressing cells in multiple sclerosis lesions, and T cells positives to EBI2 can be found in the inflamed white matter [74]. In addition, human Th17 cells are EBI2-positive and express high levels of this protein compared to other T cell subsets [74]. Therefore, EBI2 is a mediator of central nervous system autoimmunity and contributes to the migration of autoreactive T cells into inflamed organs [74].

Human Th17 cells express EBI2, and EBI2 expressing cells are abundant in the multiple sclerosis white matter lesions [74]. In addition, T cells, particularly Th17 cells expressing IL-17, have been shown to play a crucial role in multiple sclerosis pathogenesis [81].

Interestingly, Bjornevik et al., (2022) have suggested that the Epstein–Barr virus infection could be linked to the development of multiple sclerosis. This finding could be related to the increased expression of EBI2 observed in this disease [122].

#### 6.1.2. Alzheimer’s Disease

Alterations in oxysterol synthesis have been associated with neurodegenerative diseases. The *CH25H* polymorphism has been linked to Alzheimer’s disease [81]. The increased expression of *CH25H* in temporal lobe regions of the brain correlates with Braak (NFT) staging of the progression of Alzheimer’s disease [29].

In contrast to 24-hydroxycholesetrol, which is considered a protective factor to Alzheimer’s disease, 27-hydroxycholesterol is considered to be a risk factor and is increased in the cerebrospinal fluid and blood plasma of early-onset Alzheimer’s disease [123]. The release of 27-hydroxycholesterol from circulation into the brain reduces the brain glucose uptake level, Glut4 expression, and spatial memory, and can activate the renin-angiotensin system (RAS), leading to oxidative stress, impaired cognitive function, and ischemic brain injury. In addition, the RAS activation may also contribute to hypertension and insulin resistance, risk factors for Alzheimer’s disease [123].

Alzheimer’s disease, similar to the atherosclerosis process, involves macrophage infiltration, vascular occlusion, and inflammation [29]. Allelic variants in common genes, including apolipoprotein E, are involved in the predisposition to both diseases [29]. The ablation of some components of the immune system (or bone-marrow-derived macrophages alone) restricts the development of both diseases, showing the involvement of inflammatory/immune pathways [29].

Immune stimulation by pathogens, their components or autoantigens, produces local induction of CH25H in macrophages. The synthesis of 25-hydroxycholesterol confers broad-spectrum inhibition of the growth of enveloped viruses (many of them preferentially target macrophages), and potentially modulates the growth of other pathogens including parasites and intracellular bacteria [29]. The disadvantage of this anti-pathogen reaction is the chronic production of 25-hydroxycholesterol and the accumulation of cholesteryl esters via the action of acil-CoA:cholesterol aciltransferase (ACAT), the formation of fatty inclusions in macrophages, foam cell formation, and vascular occlusion [29].

### 6.2. Intestinal Diseases

Innate lymphoid cells (ILCs) are considered to be tissue-resident cells that actively migrate during inflammation [124]. ILCs include cytotoxic natural killer (NK) cells and interleukin-7 receptor alpha (CD127+) subsets, and, similar to T helper (Th) lymphocytes, can be distinguished based on signature transcription factors and effector cytokines: (a) ILC1s require the transcription factor T-BET and produce interferon-γ; (b) ILC2s express the transcription factor GATA3 and produce IL-5 and IL-13; (c) ILC3s are dependent on the transcription factor RAR-related orphan receptor gamma t (RORγt) and have the ability to produce IL-17 and/or IL-22 [124,125]. ILC3s are enriched in the intestine, where they maintain healthy tissue function by tissue repair, containment of commensal bacteria, orchestrating lymphoid-organ development, host defense, and the regulation of adaptive immunity [125].

ILC migration in inflamed tissue is controlled by the EBI2 pathway [124]. The mobilization of ILC3s from cryptopatches into the surrounding tissue occurs during intestinal inflammation. In addition, the recruitment of ILC3s to inflammatory foci in the colon is dependent on EBI2 [124]. *EBI2* can also be a gene involved in the risk of inflammatory bowel disease development. In this way, a single nucleotide polymorphism in *EBI2* increases the risk of both ulcerative colitis and Crohn’s disease [126].

Tissue injury leads to increased oxysterol synthesis, which leads to the disruption of tissue homeostasis of the immune system, initiating ILC chemotaxis and the inflammatory response [124].

Emgård et al. [125] described that ILC3s express *EBI2* and migrate to its oxysterol ligand, 7α,25-dihydroxycholesterol, produced by fibroblastic stromal cells found in intestinal lymphoid structures. They have shown that in mice lacking *EBI2* or 7α,25-dihydroxycholesterol, ILC3s failed to locate cryptopatches and isolated lymphoid follicles [125]. However, *EBI2* deficiency in ILC3s caused a defect in cryptopatches and isolated lymphoid follicles formation in the colon, but not in the small intestine [125]. In addition, localized oxysterol production by fibroblastic stromal cells provided an essential signal for colonic lymphoid tissue development, and inflammation increased the oxysterol production, causing colitis through EBI2-mediated cell recruitment. Therefore, EBI2 promotes lymphoid organ development. In addition, oxysterol-EBI2-dependent positioning within tissues controls ILC3 activity and intestinal homeostasis [125].

The high intake of 25-hydroxycholesterol was shown to alter intestinal immunity, restrain plasma cell differentiation, and compromise IgA production and the response by SREBP2 restraining, independently from the EBI2-mediated cell migration [127].

7α,25-dihydroxycholesterol is increased by inflammatory signals, and EBI2 controlled inflammatory cell recruitment during colitis. Consequently, experiments in EBI2-deficient mice demonstrate that these animals were less susceptible to colitis in an innate model of intestinal inflammation [125].

Colonic inflammation activates the oxysterol EBI2 pathway through increased production of the EBI2 ligand 7α,25-dihydroxycholesterol. In addition, there is a significant correlation between *CH25H* and *CYP7B1* expression and colonic inflammation in humans with ulcerative colitis [125].

There is much evidence that links oxysterols and inflammatory disorders such as inflammatory bowel disease. Inflammatory bowel disease can be regrouped in two frequent chronic diseases of the gut: ulcerative colitis and Crohn’s disease. The gut, where the oxysterols originated from diet, mainly from cholesterol-rich food, represents the initial site of exposure to oxysterol effects. This primary interaction, can potentially interfere with the human digestive tract homeostasis, playing a role in intestinal mucosal damage [128,129,130]. A mixture of oxysterols derived from a rich cholesterol dietary can lead to a strong pro-inflammatory effect, exhibiting cytotoxicity, apoptosis, and the development of atypical cell clones of human colonic epithelial cells. This may favor intestinal inflammation and colon cancer progression [131,132,133,134], while other oxysterols, such as 7-ketocholesterol and 25-hydroxycholesterol can decrease the vascular endothelium barrier integrity and intestinal epithelial [135].

The increased oxysterol synthesis in colon inflammation and the EBI2-7α,25-ddihydroxycholesterol axis are involved in the generation of colonic lymphoid structures in steady state and inflammation. In addition, oxysterols promote the downregulation of the CH25H enzyme, which can be further implicated in the pathogenesis of intestinal fibrosis, and could also contribute to inflammatory bowel diseases on several aspects [136]. The role of EBI2 in IL-10 colitis points towards EBI2 as a potential drug target in inflammatory bowel disease [137].

### 6.3. Rheumatoid Arthritis

Rheumatoid arthritis is a common chronic autoimmune disease that primarily affects the joints and is characterized by inflammatory infiltration of leukocytes in the synovial compartment, and by autoantibody production [138,139].

25-Hydroxycholesterol decreases IL-10 production in Th1 cells that also contribute to rheumatoid arthritis progression. In addition, in synovial tissue there is a high expression of Ch25h mRNA in individuals that have autoantibody-positive arthralgia and that are at high risk of developing rheumatoid arthritis [140].

### 6.4. Cancer

There are studies that provide some indirect evidence for the use of oxysterols in cancer immunotherapy [141], and the LXR/oxysterols axis can be the main target [142]. In addition, some oxysterols can favor tumor progression inhibiting the antitumor immune response [32], or they can also act as putative chemokines. Upon activating the membrane G-protein-coupled receptor CXCR2, tumor oxysterols recruit neutrophils to promote an immunosuppressive milieu within the tumor microenvironment [75].

Tumoral cells produce oxysterols such as 22-hydroxycholesterol and 27-hydroxycholesterol, which can dampen the generation of antitumor immune responses by inhibiting, by a CCR7-dependent pathway, the migration of dendritic cells to draining lymph nodes and by increasing the recruitment of protumor neutrophils, both mechanisms that promote tumor growth [142].

27-Hydroxycholesterol is a major biochemical mediator of the metastatic effects present in hypercholesterolemia and can increases breast cancer metastasis [123,143] through its interaction with γδ-T cells and polymorphonuclear neutrophils, probably by action on ERs, LXRs, and CXCR2. In addition, the inhibition of CYP27A1 could significantly reduce breast cancer colonization of the lungs in two clinically relevant animal models [143].

The inhibition of cholesterol synthesis can reduce the production of oxysterol and therefore partly abrogates LXR activation. In this way, zaragozic acids (ZA) have a strong effect on the inhibition of the squalene synthase without interfering with isoprenoid synthesis, on which many immune cells depend for motility and activation [144]. Lanterna et al. (2016) [144] demonstrate that ZA can inhibit the growth of the RMA lymphoma and the Lewis lung carcinoma (LLC) in animal model, without inducing side effects. However, tumor growth inhibition is dependent on an intact immune system. In addition, ZA promotes a marked reduction in the LXR target genes Abcg1, Mertk, Scd1 and Srebp-1c in the tumor microenvironment and potentiated the antitumor effects of active and adoptive immunotherapy, significantly prolonging the overall survival of tumor-bearing mice treated with the combo ZA and TAA-loaded dendritic cells [144]. In this way, the modulation of cholesterol/oxysterol synthesis can improve the effectiveness of immunotherapy and other cancer treatments.

#### Chronic Lymphocytic Leukemia

Chronic lymphocytic leukemia (CLL) is originated from clonal expansion of mature B cells expressing the B1 and T cell marker CD5. It is the most common lymphoid cancer in humans [145].

Human and mouse chronic lymphocytic leukemia (CLL) develop from CD5^+^ B cells. In mice, CD5^+^ B cells define the distinct B1a B cell lineage, which is characterized by the lack of germinal center development. In addition, in mice with reduced germinal center formation, there is an increase in B1a cell population [146]. EBI2 is the major mediator of follicular B cell migration in the lymphoid follicles in response to 7α,25-dihydroxycholesterol. In this way, downregulation of EBI2 is essential for germinal center formation. The upregulation of EBI2 drives B cells toward the extrafollicular areas [146].

The increase in EBI2 expression would leads to an expanded B1 cell subset and progression to chronic lymphocytic leukemia [146]. B cell-targeted expression of human EBI2 (hEBI2) in mice was shown to reduce germinal center-dependent immune responses, decreasing total IgM and IgG levels, and leads to increased proliferation and upregulation of cellular oncogenes [146]. In addition, the overexpression of hEBI2 leads to an abnormal expansion of CD5^+^ B1a B cell subset, late-onset lymphoid cancer development, and premature death [146]. These results are similar to those observed in human chronic lymphoid leukemia and have pointed towards EBI2 as being a promoter of B cell malignancies [146].

### 6.5. Atherosclerosis

Atherosclerosis is characterized by a progressive deposition of fat and inflammatory cells in the arterial wall. Innate immune pathways were shown to be involved in the beginning and progression of the atherosclerosis process [85]. A chronic and persistent inflammation in the arterial wall is involved in atherosclerotic lesion progression [85].

Macrophages have many roles in several steps of the development of atherosclerotic lesions [84,85,147]: in cholesterol accumulation, as mediators of the immune response, and as sources of secreted enzymes and growth factors [147].

In the early stages of atherosclerotic lesion development, macrophages engulf, aggregate, and modify LDL particles retained by intimal proteoglycans, or native LDL via pinocytosis. These macrophages are thereby converted into lipid-laden foam cells [84,85]. Macrophages in the arterial intima respond to cholesterol load, upregulating the expression of ABC transporters (ABCA1 and ABCG1) [84,85]. However, the capacity of the macrophages to remove the excess LDL lipid is overwhelmed if the influx of lipoprotein particles continues. This process leads to the development of the first atherosclerotic lesion stage, which is called fatty streak, and is typically rich in macrophage foam cells. Some of these foam cells undergo apoptosis and oxysterols play an important role in triggering this process [84].

The high levels of cholesterol in macrophages can lead to mitochondrial dysfunction, contributing to inflammatory stimulation in atherosclerosis [28]. In a persistent lipid deposition within the arterial wall, an induction of pro-inflammatory genes by innate immunity in macrophages occurs. In this situation, macrophages produce inflammatory mediators, such as cytokines and reactive oxygen species, leading to the recruitment and accumulation of other immune/inflammatory cells, such as T-lymphocytes, dendritic cells, and mast cells within the vascular wall, contributing to a complex lesion development [84,85].

Some oxysterols can modify the pro-inflammatory signaling by macrophages in the atherosclerotic lesion in several ways [84]. High levels of oxysterols have been reported in the atherosclerotic lesion, mainly 27-hydroxycholesterol and 7α-hydroxycholesterol [29,84]. 25-Hidroxycholesterol plays a role in foam cell formation and induces the secretion of pro-inflammatory cytokines and chemokines in monocytes/macrophages from human atherosclerotic plaque, such as IL-1, IL-6, IL-8, CCL5, and M-CSF [148]. On the other hand, under certain conditions, 25-hydroxycholesterol can suppress inflammasome activity by inducing IFN, suppressing SREBP, antagonizing inflammasomes, and inhibiting the Akt/NFκB signaling pathway [148].

### 6.6. Respiratory Diseases

#### 6.6.1. Asthma

In allergy conditions, oxidative stress occurs by the activation of immune system. Zanjani et al., (2022) [149] have demonstrated that there is an increase in the levels of 7-ketocholesterol, cholestane-3β, 5α, 6β-triol, and malondialdehyde (MDA) in the plasma of patients with allergic asthma [149].

In allergic inflammation there is a recruitment of leukocyte, and this recruitment is dependent on local production of cytokines, chemokines, and other potential mediators. In this way, oxysterol–EBI2 signaling participates in human asthma through a complex signaling cascade that involves Gα(i), cAMP, ERK, and Pin1 [56]. In addition, Shen et al., (2017) [56] have demonstrated a high elevation in eosinophil count in bronchoalveolar lavage from patients with mild asthma, even with increased levels of 25-hydroxycholesterol, 7β,27-dihydroxycholesterol, 27-hydroxycholesterol, and 7α-hydroxycholesterol. In addition. This increased level of oxysterols is linked with inflammatory cell counts such as eosinophils, neutrophils, and lymphocytes [56].

#### 6.6.2. Tuberculosis

Tuberculosis is caused by *Mycobacterium tuberculosis* (MT) infection and is responsible for many deaths worldwide [31]. MT has accompanied hominids for more than 70,000 years, and it has evolved and developed innate resistance to the human immune response [31]. In this way, MT has a powerful sterol-catabolizing system that is responsible for its virulence. Among these enzymes, the following stand out: (a) 3β-hydroxysteroid dehydrogenase (3βHSD)—oxidizing the 3β-hydroxyl moiety of cholesterol, this enzyme is used by MT to degrade cholesterol as its carbon source for growth; (b) CYP125—catalyzes the terminal hydroxylation of cholesterol and is required for the in vivo survival of MT, principally at the very first stages of infection; (c) CYP142—is a functionally redundant analog for CYP125; (d) CYP124—can metabolize cholesterol, and also hydroxylates methyl-branched lipids such as vitamin D3 and 7-dehydrocholesterol [31].

Varaksa et al., (2021) [31] have suggested that MT cholesterol metabolism is not only a source of carbon and energy, but also offers an evolutionary ability to MT to degrade immunoactive cholesterol derivatives. In addition, that 3βHSD, CYP124, CYP125, and CYP142 enzymes can bind and metabolize several human immunoactive oxysterols in vitro [31]. Goenka et al., (2017) [150] also reported the case of five unrelated infants born to consanguineous parents. Homozygous deletions of both gene CH25H and the adjacent gene lipase A, lysosomal acid type (LIPA) were present in all five children. LIPA encodes the enzyme lysosomal acid lipase, and its deficiency leads to Wolman disease in infants, which is a lysosomal storage disease characterized by the accumulation of cholesterol esters. Four of these infants were vaccinated with neonatal Bacillus Calmette–Guérin vaccine (BCG), and three of them developed local BCG abscesses. None of these infants had any other history of prolonged, unusual or recurrent infections. The absence of macrophage-derived 25-hydroxycholesterol probably causes an exacerbated response to the BCG vaccine and the abscess [150]. The activation of LXR in neutrophils is relevant in the context of MT infection as it mediates resistance to MT infection by the activation of the neutrophil/IL-17 axis [151].

It is known that 25-hydroxycholesterol is a potent bioactive lipid in both innate and adaptive immune systems and that it is produced and secreted by macrophages in response to the activation of TLRs or INF receptors in the early stages of infection. In this way, MT uses the 3βHSD enzyme to catalyze the conversion of 25-hydroxycholesterol in 25-hydroxycholest-4-en-3-one. Therefore, MT uses its enzymatic system to modulate the activity of 25-hydroxycholesterol, enabling it to interfere with the human immune response [31]. In addition, elevated levels of 25-hydroxycholesterol are reported in lungs of patients with chronic obstructive pulmonary disease, which is another condition associated with chronic infection [29].

In humans, the level of 7α,25-dihydroxycholesterol is regulated by 3β-hydroxysteroid dehydrogenase type 7 enzyme. In addition, 3βHSD present in MT might functionally mimic 3β-hydroxysteroid dehydrogenase type 7 enzyme, interfering with the oxysterol-mediated migration of immune cells [31].

#### 6.6.3. Chronic Obstructive Pulmonary Disease (COPD)

COPD is a chronic respiratory disorder that progress slowly and is characterized by an obstructive ventilatory pattern. COPD is very often linked to smoking (tobacco) and can lead to chronic respiratory failure. Example of COPD diseases are chronic bronchitis, chronic respiratory failure, and emphysema [152]. Kikuchi et al., (2012) [153] demonstrated an elevation in the expression of CYP27A1 in the lung of COPD patients, even at high levels of 27-hydroxycholesterol in the sputum of their patients. They have also demonstrated that the degree of 27-hydroxycholesterol production in the sputum was negatively correlated with lung function [153]. 27-hydroxycholesterol are also linked with the differentiation of lung fibroblasts into myofibroblasts and the production of extracellular matrix protein through the activation of NF-κB and upregulation of TGF- β1 [153].

27-Hydroxycholesterol is also involved in cellular senescence in the COPD pathogenesis. The expression of proteins associated with senescence was enhanced in lung fibroblasts of COPD patients treated in vitro with 27-hydroxycholesterol. In addition, the expression of CYP27A1 is upregulated in lung fibroblasts and alveolar macrophages in these patients [154].

Inducible bronchus-associated lymphoid tissue has been associated with COPD progression [155]. Patients with COPD show upregulated expression of CH25H and CYP7B1 in airway epithelial cells, regulating the inducible bronchus-associated lymphoid tissue formation with B cell migration. In this way, 7α,25-dihydroxycholesterol is the major EBI2 ligand linked to inducible bronchus-associated lymphoid tissue generation [155].

There is upregulation of CH25H localized in alveolar macrophages and pneumocytes of COPD patients. In addition, COPD patients show increased levels of 25-hidroxycholesteol in sputum that is correlated with sputum interleukin-8 levels and neutrophil counts [156]. 25-Hydroxycholesterol levels are inversely correlated with the forced expiratory volume in 1 s, percentage of predicted forced vital capacity, and diffusing capacity of carbon monoxide [156].

#### 6.6.4. Acute Lung Injury (ALI)

ALI is an acute systemic inflammatory process in lungs, which is clinically characterized by pulmonary infiltrates, hypoxemia, and edema. ALI occurs predominantly in young, previously healthy people, and causes a long-term illness and disability burden on the individual. In addition, ALI is responsible for thousands of adult and pediatric deaths worldwide every year [157].

25-Hydroxycholesterol has been reported for its action as an inflammation regulator. In this way, Ouyang et al., (2018) [158] have demonstrated that C57BL/6 mice intraperitonially pretreated with 25-hydroxycholesterol have shown improved survival rates, attenuated pathological changes in the lung, and a decrease in the release of inflammatory cytokines when these animals were intratracheally exposed to LPS [158]. 25-Hydroxycholesterol interferes with LPS binding to TLR4 complex and inhibits the activation of Akt/NF-κB signal pathway, but does not affect the MAPK pathway. However, 25-hydroxycholesterol has protective effects in LPS-induced ALI by directly targeting myeloid differentiation protein 2 [158].

In addition, Bottemanne et al., (2021) [72] have described 25-hydroxycholesterol as the only oxysterol with altered levels during lung inflammation in both ALI murine models studied by them. However, there were differences in 25-hydroxycholesterol levels between the models [72]. In an ALI induced by LPS, 25-hydroxycholesterol levels were increased, while most of the hallmarks of the model such as leukocyte recruitment, mRNA expression, and secretion of inflammatory cytokines were decreased following its intratracheal administration [72].

Table 1 summarizes the main oxysterols related to immune-related diseases.

## 7. Conclusions

Although several aspects of oxysterols have been studied in depth, their effects and actions with regard to immune cells and related diseases are not as well known. The various types of oxysterols, the numerous pathways, and the variety of cell types involved, certainly make these studies difficult. It is tempting to speculate that, soon, the knowledge of specific pathways that are stimulated or inhibited by oxysterols in cells of the immune system will soon identify new targets and lead to the development of new therapies for the diseases involved.

## Figures and Tables

**Table 1:** Oxysterols and related gene, receptors or enzymes involved with immune-related diseases.

Disease	Oxysterol, Gene or Enzyme	Reference
Atherosclerosis	↑ 27-hydroxycholesterol	[29,84]
	↑ 7α-hydroxycholesterol	[29,84]
	7-ketocholestrol	[159]
	25-hidroxycholesterol in foam cell formation	[148]
Alzheimer’s disease	↑ 25-hidroxycholesterol	[29]
	27-hydroxycholesterol	[123]
	↓ 24-hydroxycholesterol in advanced disease	[117]
	↑ 24-hydroxycholesterol in early disease	[117]
	↑ CH25H	[29]
	*CH25H* polymorphism	[81]
Multiple sclerosis	↓ 24-hydroxycholesterol in advanced disease	[102,116,117]
	↑ 24-hydroxycholesterol in early disease	[117]
	↓ 25-hydroxycholesterol in plasma	[119]
	↓ (25R)-26-hydroxycholesterol	[116]
	↓ 7α-hydroxycholesterol	[116]
	7-ketocholesterol released during inflammatory demyelination in the MS course	[118]
	↑ 25-hydroxycholesterol in spinal cord	[74,119]
	↑ 7α,25-dihydroxycholesterol in spinal cord	[74]
	↑ 7α,25-dihydroxycholesterol in CNS	[74]
	↑ 7α,26-dihydroxycholesterol in spinal cord	[74]
	↑ 7α,24-dihydroxycholesterol in spinal cord	[74]
	↓ 26-hydroxycholesterol in spinal cord	[74]
	↑ CH25H in microglia	[74]
	↑ CYP7B1 in CNS-infiltrating immune cells	[74]
	Genetic variants of *NR1H3* (LXRα)	[121]
	Genetic variants of *CH25H*	[121]
Tuberculosis	25-hydroxycholesterol modulation	[31]
	3βHSD	[31]
	CYP125	[31]
	CYP142	[31]
	CYP124	[31]
	*CH25H*	[151]
	LIPA	[151]
Chronic obstructive pulmonary disease	↑ 25-hydroxycholesterol	[29]
Intestinal diseases	High ingestion of of 25-hydroxycholesterol	[127]
Inflammatory bowel diseases	Oxysterols originated from diet	[128,129,130,131,132,133,134]
	7-ketocholesterol	[135]
	25-hydroxycholesterol	[135]
	↓ CH25H enzyme	[136]
Ulcerative colitis	*CH25H*	[125]
	*CYP7B1*	[125]
Rheumatoid arthritis	25-hydroxycholesterol	[140]
Diabetes mellitus	↑ 7α-hydroperoxycholest-5-en-3β-ol in the kidney, heart, and liver	[160]
	↑ 7β-hydroperoxycholest-5-en-3β-ol in the kidney, heart, and liver	[160]
	↑ 7α-hydroxycholesterol in the kidney, heart, and liver	[160]
	↑ 7β-hydroxycholesterol in the kidney, heart, and liver	[160]
	↑ 7-ketocholesterol in the kidney, heart, and liver	[160]
	↑ Total oxysterols in plasma	[161]
Cancer	22-hydroxycholesterol	[142]
	27-hydroxycholesterol	[123,142,143]
	LXR/oxysterols axis	[142]
	CYP27A1	[143]
Asthma	↑ 7-Ketocholesterol in plasma level	[149]
	↑ Cholestane-3β, 5α, 6β-triol in plasma level	[149]
	↑ 25-hydroxycholesterol in BLA	[56]
	↑ 7β,27-dihydroxycholesterol in BLA	[56]
	↑ 27-hydroxycholesterol in BLA	[56]
	↑ 7α-hydroxycholesterol in BLA	[56]
Chronic obstructive pulmonary disease	↑ CYP27A1 in the lung	[153]
	↑ 27-Hydroxycholesterol in sputum	[153]
	27-Hydroxycholesterol—differentiation of lung fibroblasts into myofibroblasts	[153]
	↑ CYP27A1 in lung fibroblasts and alveolar macrophages	[154]
	↑ CH25H in airway epithelial cells	[155]
	↑ CYP7B1 airway epithelial cells	[155]
	7α,25-dihydroxycholesterol linked with inducible bronchus-associated lymphoid tissue generation	[155]
	↑ CH25H localized in alveolar macrophages and pneumocytes	[156]
	↑ 25-Hidroxycholesteol in sputum	[156]
Acute lung injury	25-Hydroxycholesterol protection from ALI	[158]
	25-Hydroxycholesterol altered levels during lung inflammation in ALI	[72]

↑ increase or upregulation; ↓ decrease or downregulation; CH25H—Cholesterol 25-Hydroxylase; NR1H3—Nuclear Receptor Subfamily 1 Group H Member 3; CYP7B1—Cytochrome P450 Family 7 Subfamily B Member 1; 3βHSD—3β-hydroxysteroid dehydrogenase; CYP125—Mycobacterial cytochrome p450 125; CYP142—Mycobacterial cytochrome P450 monooxygenase 142; CYP124—Mycobacterial cytochrome P450 124; LIPA—lipase A; CYP27A1—Cytochrome P450 Family 27 Subfamily A Member 1; CNS—central nervous system; MS—multiple sclerosis, BLA—bronchoalveolar lavage, ALI—Acute lung injury.

## Data Availability

Not applicable.
